# Oncolytic Viruses and Immune Checkpoint Inhibitors: Preclinical Developments to Clinical Trials

**DOI:** 10.3390/ijms21228627

**Published:** 2020-11-16

**Authors:** June Kyu Hwang, JinWoo Hong, Chae-Ok Yun

**Affiliations:** 1 Department of Bioengineering, College of Engineering, Hanyang University, 222 Wangsimni-ro, Seongdong-gu, Seoul 04763, Korea; psients@gmail.com (J.K.H.); jhong803@gmail.com (J.H.); 2 GeneMedicine Co., Ltd., 222 Wangsimni-ro, Seongdong-gu, Seoul 04763, Korea; 3 Institute of Nano Science and Technology, 222 Wangsimni-ro, Seongdong-gu, Seoul 04763, Korea

**Keywords:** oncolytic virus 1, immune checkpoint inhibitor 2, immuno-oncology 3, combination therapy 4

## Abstract

Immuno-oncology (IO) has been an active area of oncology research. Following US FDA approval of the first immune checkpoint inhibitor (ICI), ipilimumab (human IgG1 k anti-CTLA-4 monoclonal antibody), in 2011, and of the first oncolytic virus, Imlygic (talimogene laherparepvec), in 2015, there has been renewed interest in IO. In the past decade, ICIs have changed the treatment paradigm for many cancers by enabling better therapeutic control, resuming immune surveillance, suppressing tumor immunosuppression, and restoring antitumor immune function. However, ICI therapies are effective only in a small subset of patients and show limited therapeutic potential due to their inability to demonstrate efficacy in ‘cold’ or unresponsive tumor microenvironments (TMEs). Relatedly, oncolytic viruses (OVs) have been shown to induce antitumor immune responses, augment the efficacy of existing cancer treatments, and reform unresponsive TME to turn ‘cold’ tumors ‘hot,’ increasing their susceptibility to checkpoint blockade immunotherapies. For this reason, OVs serve as ideal complements to ICIs, and multiple preclinical studies and clinical trials are demonstrating their combined therapeutic efficacy. This review will discuss the merits and limitations of OVs and ICIs as monotherapy then progress onto the preclinical rationale and the results of clinical trials of key combination therapies.

## 1. Introduction

In past decades, cancer treatment has made tremendous progress to include a range of therapies. One specific therapeutic area, immuno-oncology (IO), has received much renewed attention for its ability to take advantage of the body’s immune system to fight cancer. Specifically, the development of antibodies (Ab) that target key immune checkpoint molecules served as a revolutionary milestone in the field of IO, as the Abs were able to disrupt the cancer cells’ ability to evade the host immune response and activate anti-tumor immunity [1,2]. Such developments led to the first US FDA-approved immune checkpoint inhibitor (ICI), ipilimumab—a cytotoxic T-lymphocyte antigen-4 (CTLA-4)-targeting monoclonal Ab—for the treatment of patients with advanced melanoma [1]. After approval of ipilimumab, several other immune checkpoint blockade agents were examined, leading to numerous clinical trials that examined the efficacy of ICIs for either mono or combination therapy. To date, six other ICIs have been approved by the US FDA, comprising programmed death 1 (PD-1) inhibitors (pembrolizumab, nivolumab, and cemiplimab) and programmed death-ligand 1 (PD-L1) inhibitors (avelumab, durvalumab, and atezolizumab) [1]. Overall, the increase in ICIs is part of the growing IO trend: between 2017 and 2019, Tang et al. found a 91% increase in the number of active agents, a 78% increase in active IO targets, and a 60% increase in participating IO drug development organizations, resulting in 31 IO drug approvals by the US FDA [3].

Despite the impact of ICIs in IO and their potential in the clinical setting, accumulating evidence suggests that researchers must overcome some critical limitations: (a) some patients undergoing ICI therapy experience severe immune-related adverse events (irAE) [4], (b) only a fraction of cancer patients benefit from ICI treatment, and c) ICIs are ineffective against immunologically ‘cold’ tumors characterized by a low tumor infiltrating lymphocyte (TIL) count [5]. To this end, oncolytic viruses (OV), which preferentially infect and lyse cancer cells, have been proposed as a promising modality for combination therapy to address the limitations of ICI. This is due to the unique ability of OVs to inflame a “cold” tumor microenvironment (TME) into a “hot” environment with increased immune cell and lymphocyte infiltration, making them an ideal candidate for combination with various cancer immunotherapeutics [5]. For this reason, multiple ongoing clinical trials are aiming to investigate the combined therapeutic efficacy of ICIs and OVs. This review will discuss the underlying rationale behind ICIs and OVs as a synergistic combination therapy regimen and their application in preclinical and clinical settings (Table 1: List of Ongoing Combination Clinical Trials with Oncolytic Viruses and Immune Checkpoint Inhibitors).

## 2. Immune Checkpoint Inhibitors

Immune checkpoints are regulators of the immune system that are crucial for self-tolerance and prevention of the immune system from attacking cells indiscriminately [6]. Many tumors evade the host immune system by upregulating immune checkpoints to generate immunosuppressive TME [7]. ICIs work by disrupting such tumor immunosuppressive signaling pathways, exposing the cancer cells to the host immune system. There has been overwhelming preclinical evidence validating PD-1, PD-L1 and CTLA-4 inhibitors as viable targets for IO therapy, with demonstrated clinical efficacy against several solid and hematologic malignancies [1]. To date, six US FDA-approved drugs, comprised of three PD-1 inhibitors (nivolumab, pembrolizumab, and cemiplimab), three PD-L1 inhibitors (atezolizumab, avelumab, and durvalumab) and two CTLA-4 inhibitors, are used in the clinical setting [1,8]. In addition, new emerging checkpoint inhibitors targeting lymphocyte-activation gene 3 (LAG-3) [9,10] or T cell immunoglobulin and mucin-domain containing-3 (TIM-3) [11] have demonstrated promising preclinical results and in clinical development.

Although ICIs have had an unprecedented effect on the clinical activity of several cancers to transform IO, ICIs—both as mono and combination therapy—are not without limitations. Primarily, ICIs are known to have a broad range of side effects affecting every organ system, as well as producing irAE in a significant incidence of patients [12]. Flaherty et al. identified longer follow-ups of clinical trial population studies to accumulate evidence of late relapses, suggesting an emergence of acquired resistance to ICIs [13]. There are several interesting targets, like pattern recognition receptors, that were successfully exploited in preclinical models to circumvent resistance to T cell-targeted ICIs, but these investigations are in early stages and none have reached the clinical development stage [14]. In addition, several groups have reported that a diversity of neoantigens may be necessary to induce a sufficient clinical response from ICIs [15], with van Rooij et al. [16] and Le et al. [17] observing neoantigen-specific T cell reactivity in patients with advanced cancers. Moreover, several groups have substantiated the role of neoantigens in response to ICIs, supported by evidence of higher neoantigen load being associated with better therapeutic response to CTLA-4 and PD-1 blockade in patients with melanoma and non-small-lung cancer [18,19,20]. However, ICIs are most critically limited by their refractoriness or ineffectiveness in ‘cold’ TME, as there is a lack of T cell infiltration, resulting in overall suppression of host antitumor immune response [5]. Furthermore, as many types of cancers present with ‘cold’ tumors, ICIs are critically limited in therapeutic potential, necessitating the identification of novel IO candidates as combination therapies to maximize the clinical benefits of individual ICIs.

### Development of Oncolytic Viruses in Combination with Immune Checkpoint Inhibitors

Oncolytic viruses (OVs) are native or recombinant viruses that self-propagate, selectively replicate in cancer cells, and infect adjacent cancer cells to elicit a cytolytic effect. Specifically, OVs differ from conventional cancer therapies in that they have naturally evolved to effectively hijack and reprogram the host’s cellular machinery, effectively expressing both virus and therapeutic transgenes at a high level [21]. OVs have been demonstrated to stimulate the immune system by infecting a tumor cell to induce immunogenic cell death, which triggers an inflammatory reaction. This particular form of apoptosis results in the release of immune stimulatory agents, which activate innate and direct adaptive immune responses against cancer cells by the release of pathogen-associated molecular patterns (PAMPS), tumor-associated antigens (TAAs), and danger-associated molecular patterns (DAMPS) from lysed tumor cells [22,23]. APCs in the TME then recognize these key metabolites and generate an immune response. Furthermore, this local stimulation of the immune system creates a systemic and enduring anti-cancer response [24]. Importantly, these attributes of OVs have been shown to revert immunologically ‘cold’ or ‘non-inflamed’ tumors with low TIL count into ‘hot’ tumors, indicating them as promising candidates to synergize with cancer immunotherapeutics [24,25,26]. There is a strong line of evidence that suggests viruses, like rotavirus, influenza or yellow fever virus, can also prime TME of cold tumors to be more responsive toward immunotherapy [27]. In particular, efforts to synergize OVs with other IO drugs have demonstrated OV infections to increase the expression levels of several proinflammatory cytokines and cause an influx of natural killer cells (NKs), activated T cells, and APCs into tumor tissues [28], priming unresponsive TMEs into more conducive targets for other cancer immunotherapeutics.

OVs provide additional versatility as effective anticancer agents, as they can be genetically engineered to possess specificity toward tumor cells and to preferentially lyse malignant cells, focusing their therapeutic transgene delivery and simultaneously reducing potential off-target side effects [29]. Importantly, arming OVs with antitumor immune transgenes, like cytokines, can lead to localized and high expression levels of these genes in tumor tissues, with minimal systemic circulation. These attributes allow OVs to deliver potent immune stimulators, like IL-12 [30] and 4-1BBL [31], among others, which had failed in clinical development as protein therapeutics due to their high systemic toxicity [32]. Despite these remarkable advances in OV IO, these viruses must overcome certain challenges to achieve clinical application and commercialization. Namely, most early clinical trials that administered OVs intratumorally as a monotherapy were not able to demonstrate a sufficient therapeutic outcome [33,34,35]. OVs have been shown to be susceptible to selective pressure of heterogeneous tumor populations when administered over an extended period of time, as these viruses lose essential signaling pathways by mutation or lose important receptors that mediate their life cycle [36].

Due to the above limitations, first-generation OVs like Rigvir and ONYX-015 have shown limited therapeutic efficacy as monotherapies, and their approval for in-human use has been restricted to the countries (Latvia and China, respectively) that commercialized these products [37,38]. Rigvir, a non-pathogenic ECHO-7 virus with no genetic modifications, was the first OV approved by regulatory authorities, gaining approval in Latvia in 2004 for the treatment of melanoma [37]. However, Rigvir did not achieve global commercialization; most of its clinical studies were performed before 1991, its safety and efficacy parameters needed to be updated to modern standards, and its response rate needed to be increased by optimizing its biomarkers toward more specific target populations [37]. Since Rigvir, several other OVs, which are innately oncolytic without genetic modification of the virus genome, have entered clinical development in the US and EU [39]. Still, most first-generation OVs, such as Oncorine (previously designated as ONYX-015) and HSV1716, which began clinical trials in the 1990s, as well as those that are currently under clinical evaluation, harbor several genetic modifications to improve the cancer specificity of virus strains. The first such genetically modified OV to be approved by a regulatory authority was Oncorine, which was approved by the Chinese FDA in 2005 for the treatment of solid tumors [40]. Despite gaining Chinese FDA approval, Oncorine’s development in the US and EU failed to induce significant tumor growth inhibition in patients when used as a monotherapy and only achieved meaningful clinical therapeutic responses when used in combination with chemotherapy [41] or radiotherapy [42], demonstrating its inadequacies and suboptimal potency as a monotherapy.

Since commercialization of Rigvir and Oncorine in the early 2000s, only one other OV, Imlygic, has been approved for human use. Imlygic (granulocyte-macrophage colony-stimulating factor (GM-CSF)-armed, herpes simplex virus type 1 (HSV10)), which was approved by the US and EU FDAs in 2015 and by the AUS FDA in 2016 [43,44], remains the only internationally available OV with a proven clinical track record as a monotherapy against melanoma [45]. Unlike Rigvir and Oncorine, developed in the 1990s, which were primarily evaluated in a clinical setting for direct oncolytic effects, Imlygic was designed to maximize its therapeutic potential as a next-generation IO therapeutic, armed with antitumor immune-boosting transgenes like GM-CSF, and clinically examined with a strong emphasis on immune-related evaluation parameters, such as immune cell infiltration into tumor tissues and cytokine profiling. In general, this IO-oriented clinical development process of Imlygic outlines the drastic change in the perception of OVs as cancer immunotherapeutics in the modern cancer therapy landscape. There is a growing amount of evidence supporting this IO-driven development process: for instance, several studies have shown that OVs exerting minimal cytolytic effect against tumors can effectively suppress the growth of injected and non-injected distal tumors by inducing an antitumor immune response [46]. The landmark approval of Imlygic has accelerated clinical development of other OVs. Imlygic has been evaluated in the largest number of ongoing and completed clinical trials for any particular OV, as either a monotherapy or a combination therapy, and has generated large quantities of clinical data [47,48]. Such trials have demonstrated that OVs can inflame the immunosuppressed TMEs of clinical tumors and have provided strong empirical evidence for combining ICIs with OVs [23,49,50]. A positive phase II clinical trial results from “Ipilimumab With or Without Imlygic in Unresected Melanoma (NCT01740297)” was the first to demonstrate that synergistic therapeutic efficacy can be achieved with combination of OV and ICI in cancer patients, as a higher level of cytotoxic T cell infiltration in tumors was observed following combination treatment [47] compared to that following ipilimumab monotherapy [47]. Notably, the combination of Imlygic and ipilimumab did not induce more severe side effects than monotherapy, whereas ipilimumab in combination with other ICIs has been reported to elevate the incidence rates and severities of adverse events [51]. Not only does this evidence support OVs as a viable and effective complementary therapy strategy in combination with ICIs, but it also sets a precedent for future combination therapies for clinical development, which are discussed in detail below.

## 3. Oncolytic Viruses in Combination with Immune Checkpoint Inhibitors in Clinical Trials

### 3.1. Adenovirus

Adenoviruses (Ads) are non-enveloped and double-stranded DNA viruses [52] with an icosahedral capsid. These viruses range from 70–90 nm in size and possess a genome of approximately 35 kb [53]. While 57 known Ad serotypes exist, serotype 5 is the most commonly used backbone in OVs [54]. Ads are one of the most commonly used viral vectors in cancer gene therapy due to their advantageous characteristics, which include high gene transfer efficiency in both dividing and non-dividing cells, low risk of insertional mutagenesis, and exponential viral replication capacity (one infectious virus can produce more than 10,000 progeny viruses in an infected cancer cell) [21,45,55,56,57]. Furthermore, Ads have been shown to elevate the expression level of immunostimulatory signals to enhance TAA presentation by APC to induce a strong, tumor-specific immune response [12], illustrating them as one of the most immunogenic OVs [58]. For this reason, several oncolytic Ads are under evaluation in clinical trials as monotherapies and in combination with ICIs.

#### 3.1.1. Tasadenoturev (DNX-2401) + Pembrolizumab

DNX2401 is an oncolytic Ad with improved infectivity and tumor selectivity [59], achieved by a 24-bp deletion in the Rb binding site of the E1A region, which enables selective replication in cancer cells that lack a functional Rb pathway [60]. With positive preclinical evaluation studies [61], DNX2401 is currently undergoing three clinical trials as a monotherapy (NCT03714334, NCT03896568, and NCT03178032) for treatment of malignant gliomas. While the vector showed tumor size reductions in 72% of patients following intratumoral injection, with a median overall survival time of 9.5 months [62]. More relevant to the scope of this review is that Lang et al. found decreased TIM-3 expression through immunohistochemical analysis of resected tumor specimens [63]. TIM-3 is an immune inhibitory receptor that is known to promote T cell exhaustion [62], and the author suggests that DNX-2401 infection may overcome some aspects of the T cell exhaustion of glioma patients, providing a rationale to investigate the vector in combination with anti-PD-1 Ab [63]. A phase II trial is currently ongoing to investigate intratumorally administered DNX-2401, followed by the PD-1 inhibitor pembrolizumab in RGBM patients (NCT02798406). Interim results have been reported for 48 subjects who received the combination of pembrolizumab and DNX-2401 and achieved a median overall survival of 12 months; 47% experienced a clinical benefit (stable disease or better). In detail, four subjects had partial responses, two of whom showed 94% regression of tumor volume and three survived for more than 20 months [64].

#### 3.1.2. ONCOS-102 (Ad 5/3 Δ24 GM-CSF) + Pembrolizumab

ONCOS-102 is a highly modified oncolytic Ad vector including a chimeric 5/3 fiber-knob region for increased infectivity, a 24-bp deletion in the E1A region for selective replication in cells with dysfunctional Rb pathway, and the transgene insertion of GM-CSF to improve immune cell infiltration at the tumor site [65,66]. ONCOS-102 has been extensively tested in preclinical investigations [65] and has progressed to a phase I clinical trial (NCT03003676) in combination with the PD-1 ICI pembrolizumab. This phase I trial, which treated 12 patients with advanced or unresectable solid tumors, concluded ONCOS-102 to be safe and well tolerated at the tested doses, and combination therapy induced significant immune cell infiltration at the tumor sites [66]. The results of the study concluded that the combination therapy produced 5.9- and 4.0-fold increases in infiltration of CD3+ immune cells and CD8+ T cells, respectively, in post-treatment tumor biopsies compared to pre-treatment samples [66]. Interestingly, 2 of the 12 patients demonstrated increased PD-L1 expression following ONCOS-102 administration and developed systemic anti-tumor immunity, made evident by melanoma associated antigen 3 (MAGE-A3)-specific CD8^+^ T cells and New York esophageal squamous cell carcinoma 1(NY-ESO-1)-specific CD8^+^ T cells [67,68]. Following these observations, ONCOS-102 has motivated another phase I clinical trial (NCT03003676), which aims to utilize ONCOS-102 with pembrolizumab in recurrent patients after PD1 blockade.

#### 3.1.3. Enadenotucirev + Nivolumab

Enadenotucirev is an Ad11p/Ad3 chimeric oncolytic Ad that was shown to elicit potent antitumor efficacy in orthotopic human tumor xenograft models [69,70]. To date, two phase I clinical studies have been conducted to assess the safety of the virus, expanding upon preclinical evidence supporting enadenotucirev’s uniqueness among OVs, namely, its superior stability in the bloodstream following intravenous delivery [63,71,72], and the low prevalence of neutralizing Ab against enadenotucirev in the general population [73,74]. The results from the first trial revealed that intravenous infusion of enadenotucirev in patients with epithelial tumors can induce infiltration of a large number of CD8^+^ T cells into most tumor samples [63] and provided a rationale to investigate the virus in combination with an anti-PD-1 Ab, nivolumab, in patients with epithelial carcinoma (NCT02636036). Currently, NCT02636036 is actively recruiting trial participants.

#### 3.1.4. Other Adenoviruses

More Ads are expected to enter clinical trials as complementary therapies with ICIs due to their strong immunostimulatory properties [75,76]. TILT-123, an oAd armed with two potent anti-tumor cytokines (tumor necrosis factor [TNF]-alpha and IL-2) that promote immune cell recruitment to TMEs, is one of the candidates that is expected to enter a phase I clinical trial by 2021, in combination with the anti-PD-L1 ICI avelumab [77]. This is based on strong preclinical evidence of TILT-123 in combination with an anti-PD-L1 ICI exerting a strong antitumor immune response in patient-derived urological tumor histocultures, as evidenced by the high expression levels of antitumor cytokines and T cell trafficking chemokine CXCL10 that positively correlated with increased intratumoral infiltration of CD8+ T cells [78]. The combination therapy led to complete regression of tumors and superior survival rate in comparison to either ICI or OV monotherapy in an animal model, showing that the immune stimulatory property of oncolytic Ad could achieve promising therapeutic outcomes in combination with PD-L1-targeted ICIs [79].

Two other oncolytic Ads have recently entered clinical trials as combination therapies with ICIs, and this continuous increase in the number of clinical investigations highlights the promising nature of this strategy. In detail, CG0070, a GM-CSF-armed serotype 5 oAd [80,81], entered phase II clinical trial in combination with pembrolizumab (NCT04387461) in May 2020. The study is expected to evaluate the activity of CG0070 through intravesical administration along with intravenous administration of pembrolizumab in patients with non-muscular invasive bladder cancer. Another virus, OBP-301, a serotype 5 oAd replicating under the control of a human telomerase reverse transcriptase promoter [82], began a phase I trial in combination with a PD-1 inhibitor, pembrolizumab (NCT03172819), for the treatment of advanced solid tumors.

As an alternative to combination therapy, where an ICI and OV are sequentially administered, several groups have investigated the insertion of ICIs, such as 4-1BBL [83] and miniaturized Ab against anti-PD1 or anti-CTLA-4, into oAd as therapeutic transgenes [84]. To date, Hemminki et al. have developed the first Ad to produce a full-length CTLA-4 monoclonal Ab (Ad 5/3 Δ24a CTLA4) while retaining the oncolytic potency of the virus, overcoming the limitations of the CTLA-4 blockade with monoclonal Ab, which has been shown to inhibit viral replication [85]. In addition, the author demonstrated effective induction of T cell activity in in vitro and in vivo models in normal donors and in patients with advanced solid tumors [86]. Meanwhile, Du et al. have reported the generation of an oAd (SKL002) with an anti-CTLA4 Ab gene inserted into the E3 region of the viral genome with anti-tumor efficacy in both in vitro and in vivo models [85].

### 3.2. Herpes Simplex Virus-1

HSV is a virus characterized by an icosahedral capsid, approximately 200 nm in size, and a linear, 150-kb double-stranded DNA genome [87,88]. Oncolytic HSV (oHSV) has been extensively used in clinical immunotherapy and is considered a useful oncolytic vector due to its large transgene capacity, lack of insertional mutagenesis, and ability to activate innate and adaptive immune responses against tumors [89]. Furthermore, HSV infection can be controlled by well-established antiviral drug regimens, ensuring greater safety in the case of a vector that threatens the life of the patient [90,91]. To date, various strains of HSV are used as monotherapies and have successfully expressed various transgenes, such as p53 [92], IL-2 [93,94], and IFN-γ [95], in preclinical studies. Currently, Imlygic remains the only US FDA-approved OV, but several other oHSVs have progressed to phase II/III clinical trials.

#### 3.2.1. Imlygic + Ipilimumab and Imlygic + Pembrolizumab

Imlygic is the first and only US FDA-approved OV and includes GM-CSF and deletion in the ICP34.5 gene to augment the tumor specificity of the virus [96,97]. Imlygic is the most extensively characterized OV in clinical settings. During the landmark OPTiM phase III trial, which subsequently led to commercialization of Imlygic, intralesional injection of the virus in patients with metastatic melanoma was shown to change the immune cell makeup of the TME into a immunologically ‘hot’ environment, as evidenced by decreases in immunosuppressive cell populations (Tregs, CD8+ T suppressor cells, and myeloid-derived suppressor cells) [98]. In addition, Imlygic increased the local and systemic T cell-recognized melanoma antigen (MART)-1-specific CD8+ effector cell populations, demonstrating the establishment of tumor-specific adaptive immunity by OV treatment [99].

These immune stimulatory properties of Imlygic and the inflammation of TME observed during the monotherapy trials provided a strong rationale for subsequent clinical investigation of the virus in combination with ICIs. A phase Ib trial investigating Imlygic in combination with a CTLA-4 inhibitor, ipilimumab, was conducted in patients with untreated late stage melanoma and achieved positive results [100]. In detail, the combination therapy led to an objective response rate and a durable response rate of 50% and 44%, respectively [101], and there were no dose-limiting toxicities (DLTs) or compounding overall adverse events compared to those of ipilimumab monotherapy [101]. In a subsequent phase II trial, 39% of patients in the combination group (Imlygic + ipilimumab) had an objective response, while only 18% of patients in the ipilimumab monotherapy arm responded. Furthermore, an abscopal response, a decrease in non-injected visceral lesions, was observed in 52% of patients in the combination group compared to 23% in the ipilimumab group [47]. These results confirmed that the combination therapy can elicit synergistic and systemic antitumor effects against both virus-injected tumors and distant non-injected metastases.

Imlygic also has been investigated as a combination therapy with the anti-PD-1 Ab pembrolizumab for treatment of melanoma, with promising results. The interim results of the MASTERKEY-265/KEYNOTE-034 phase Ib/III trial (NCT02263508) revealed that the combination therapy resulted in an objective response rate of 62%, with a complete response rate of 33%, no DLT, increased intratumoral expression level of PD-L1, and increased amounts of circulating T cells, inflaming both the virus-injected and non-injected distal tumor sites [23]. While this clinical trial is ongoing, anticipated results from MASTERKEY-265/KEYNOTE-034 have set a precedent for other clinical investigations combining ICIs with Imlygic (Table 1).

#### 3.2.2. C-REV + Ipilimumab and C-REV + Nivolumab

C-REV (formerly HF-10) is an oHSV derived from spontaneous mutation and does not express any therapeutic transgenes [102]. In preclinical studies, C-REV replicated effectively within tumors and induced an antitumor immune response, as evidenced by an increase in the numbers of activated CD4^+^, CD8^+^ T, and NK cells in tumor tissues, leading to prolonged survival and a significant reduction in tumor growth [102]. With strong preclinical evidence of a robust antitumor immune response, C-REV progressed to a phase II clinical trial as a combination therapy with the CTLA-4 inhibitor ipilimumab for treatment of the late stages of unresectable melanoma (NCT02272855).

Following the success of this clinical trial, two recent clinical trials have set out to investigate C-REV as a combination therapy with a CTLA-4 inhibitor, ipilimumab (NCT03153085), in Japanese patients with unresectable or metastatic melanoma or with a PD-1 inhibitor, nivolumab (NCT03259425), in patients with resectable stage IIIB, IIIC, or IVM1a melanoma. In the interim report of the NCT03259425 trial, patients receiving the combination of C-REV and ipilimumab demonstrated higher T cell infiltration into tumors than those achieved with the respective monotherapies in previous clinical trials [103], lower levels of PD-L1 on monocytes after 2 months of treatment, and persistent C-REV infection in 5 of 11 patients [104]. The combination of C-REV and ipilimumab was shown to exert potent anti-tumor activity, with an overall response rate and disease control rate of 41% and 68%, respectively, which were higher than the 4% and 16% achieved by ipilumab monotherapy from a trial in Japanese patients with melanoma [105]. The addition of C-REV therapy did not exacerbate the toxicity of ipilumab, as similar rates of grade 3 or higher adverse events were observed in the combination therapy trial to those observed with the previous trial evaluating ipilumab monotherapy in melanoma patients.

### 3.3. Vaccinia Virus

Vaccinia virus is a member of the *Poxviridae* family and possesses a large double-stranded DNA of 190 kb, indicating its suitability for large transgene insertion [106]. Furthermore, vaccinia virus replicates in the cytoplasm, eliminating the risk of insertional mutagenesis [53]. In addition, vaccinia viruses have a rapid replication and infection cycle that starts as early as 2 h after infection, with cell lysis taking place between 12 and 48 h [107]. For these reasons, vaccinia viruses are useful in the development of oncolytic virotherapy.

#### Pexa-Vec + Nivolumab and Pexa-Vec + Durvalumab or Pexa-Vec + Durvalumab + Tremelimumab and Pexa-Vec + Ipilimumab

Pexa-Vec is an oncolytic vaccinia virus with a thymidine kinase gene deletion that expresses GM-CSF and β-galactosidase as transgenes [107,108,109]. Pexa-Vec has been demonstrated to be capable of targeting and infecting tumor-associated endothelial cells [110]. In a preclinical setting, Pexa-Vec intravenous administration prior to surgical resection in patients with advanced solid tumors was shown to activate NK cells as well as CD4^+^ and CD8^+^ T cells in tumor tissues, in addition to elevating the expression levels of antitumor immune cytokines including IFN-α, IL-3, IL-12p40, IL-16, and IL-18 [110]. A phase Ib trial of intravenously administered Pexa-Vec for the treatment of colorectal cancer revealed that systemically administered virus can be trafficked to the tumor, as evidenced by a viral genome in tumor biopsies from seven of eight evaluable patients [107]. Furthermore, systemic administration of Pexa-Vec in patients with solid tumors prior to surgical resection was reported to induce robust activation of NK, CD14+ APC, CD4+, and CD8+ T cells in peripheral blood mononuclear cells, showing evidence of antitumor immune response induction [111,112]. Based on these encouraging antitumor immunostimulatory properties of Pexa-Vec as a monotherapy, Pexa-Vec in combination with a PD-1 inhibitor, nivolumab, is being evaluated as a first-line therapy in a phase I/II clinical trial in patients with renal carcinoma (NCT03071094) [89], which has no published results available to date.

Pexa-Vec is being investigated in another phase I/II trial with the PD-1 inhibitor durvalumab, as well the CTLA-4 inhibitor tremelimumab, in 35 patients with refractory colorectal cancer (NCT03206073). Specifically, two doses of Pexa-Vec were injected intravenously four times together with repeated infusions of durvalumab (group A) or a combination of durvalumab and tremelimumab (group B) [113]. Although no interim results of the clinical trials are available, a preclinical evaluation of the same combination therapy regimens in murine renal carcinoma tumor models revealed that locally administered Pexa-Vec in combination with either PD-1- or CTLA-4-targeted ICI resulted in superior tumor growth inhibition compared to monotherapy with either the virus or the respective ICIs in both the virus-injected primary tumor and the non-injected distal tumors. This response was due to increased CD4+ and CD8^+^ T cell infiltration into the tumor tissues [114]. Intriguingly, systemic administration of Pexa-Vec in combination with a PD-1 inhibitor led to a smaller quantity of virus in tumor tissues with respect to Pexa-Vec monotherapy, whereas the virus quantity in tumors following local administration of Pexa-Vec was unaffected by PD-1 ICI combination therapy. These findings suggest that T cell activation by PD-1 ICI may hamper the therapeutic efficacy of systemic oncolytic virotherapy, and this could be one of the underlying reasons why most OV and ICI combination therapy regimens in clinical development employ local administration of OV. Importantly, the triple combination of Pexa-Vec with PD-1 and CTLA-4 ICIs induced superior tumor growth inhibition in comparison to dual ICI combination therapy (PD-1 and CTLA-4 ICI) or Pexa-Vec monotherapy, resulting in complete tumor regression in 40% of treated mice; in contrast, Pexa-Vec in combination with a single ICI failed to induce complete regression.

As ICI combination therapy regimens are under active clinical investigation, Pexa-Vec’s ability to synergize with different ICIs is a promising attribute. Unfortunately, the study did not include safety assessment of the triple combination strategy. Intratumorally administered Pexa-Vec in combination with the CTLA-4 inhibitor ipilimumab in patients with metastatic or advanced tumors is being evaluated in a phase I clinical trial (NCT02977156). The study is aiming to conduct a two-part phase I trial to define the feasibility, safety, and antitumor effects of the combination therapy and is currently recruiting participants.

### 3.4. Coxsackievirus

Coxsackievirus is a small, 30-nm-sized, non-enveloped, single-stranded RNA virus with an icosahedral capsid belonging to the *Picornaviridae* family [53]. While the virus is grouped into two subtypes, A and B, the serotype A21 has been most commonly utilized in clinical trials for its inherent cytotoxicity against cancer cells [115]. Serotypes A13, A15, and A18 are being explored for oncolytic potential [116]. Coxsackievirus A21 is a naturally occurring OV and has not been as extensively characterized as other virus strains like HSV or Ad, which have been clinically developed as OVs since the 1990s [115].

#### CVA21 (CAVATEK) + Ipilimumab and CVA21 + Pembrolizumab

CAVATEK is an unmodified coxsackievirus A21 that demonstrated oncolytic activity in preclinical investigations [23,115]. CAVATEK binds to cancer cells by interacting with intercellular adhesion molecule 1 (ICAM-1) and decay-accelerating factor (DAF) [117]. For this reason, initial preclinical investigations examined the therapeutic effects of CAVATEK against metastatic melanoma cells, which are known to overexpress ICAM-1 [118,119]. Subsequently, the phase II trial CALM, with CAVATEK as a monotherapy against advanced melanoma patients, demonstrated that the virus induced antitumor responses, as evidenced by increased expression levels of IL-8 and IFN-γ in serum samples of melanoma patients following viral administration [120]. This evidence has led to four phase I combination clinical trials involving CAVATEK: two with the CTLA-4 inhibitor ipilimumab and two with the PD-1 inhibitor pembrolizumab.

The first phase I combination trial with CAVATEK and ipilimumab in advanced melanoma patients (NCT02307149) concluded that the combination therapy was well tolerated in a total of 16 patients and induced antitumor activity in both local, visceral, and non-visceral lesions in 12 patients that had failed previous lines of immunotherapy [121]. The second phase I clinical trial, CLEVER (NCT03408587), which combines CAVATEK and ipilimumab, aims to evaluate the combination therapy for its initial response in uveal melanoma that has metastasized to the liver.

In addition, another phase I combination trial evaluating CAVATEK and pembrolizumab, CAPRA (NCT02565992), was conducted in patients with advanced melanoma. In an interim abstract of the CAPRA trial, the authors reported the combination therapy to be well tolerated, with 11 of the 14 patients showing an objective response rate of 73% and a disease control rate of 91% [122]. Furthermore, the authors noted tumor volume reductions in both injected and non-injected lesions, leading to the recruitment of additional patients (targeted patient number = 50) [122]. Similar to the findings of the CAPRA trial, an interim report of the combination phase I trial of CAVATEK and pembrolizumab, KEYNOTE-200 (NCT02043665), reported the combination therapy to be generally well tolerated, with no reported DLT in six patients with advanced solid tumors [123].

### 3.5. Reovirus

Reoviruses are non-enveloped and double-stranded RNA viruses belonging to the *Reoviridae* family with icosahedral capsids [124]. Reoviruses are 75–80 nm in size [53] and replicate in the cytoplasm [124]. The most extensively developed oncolytic strain of reovirus is Type 3 Dearing virus (Reolysin), which has been investigated in more than 30 clinical trials [125]. Reolysin selectively replicates in and exerts oncolytic effect against cancer cells with activating Ras mutation, which is observed in approximately 30% of all human cancers [126,127].

#### Reolysin (Pelareorep) + Pembrolizumab

The immunostimulatory effects of Reolysin have been well characterized by preclinical investigations, setting a rationale for combining the virus with ICIs [128,129]. In support, combination of intratumoral administration of Reolysin and systemically administered anti-PD-1 ICI resulted in significantly prolonged survival in an immunocompetent murine melanoma tumor model compared to monotherapy treatment with either compound [129]. Further, Rajani et al. reported Reolysin injection to prime tumors toward PD-1 blockade, as evidenced by an enhanced CD8^+^ T cell Th1 antitumor response compared to that after PD-1 monotherapy [129]. Samson et al. reported a similar immunostimulatory effect with reovirus infection. Compared to untreated brain tumors, those infected with a reovirus infusion prior to undergoing debulking neurosurgery demonstrated a marked increase in tumor-infiltrating CD8^+^ cells in resected immunohistochemical specimens [130].

Additionally, preclinical and clinical studies evaluating Reolysin in combination with chemotherapeutic drugs such as gemcitabine, irinotecan, or taxanes have been shown to enhance viral replication and cytolytic effects [131,132,133]. Based on these results, a phase I trial combining Reolysin with one of three chemotherapeutic drugs (5-fluorouracil, gemcitabine, or irnotecan) followed by administration of the PD-1 inhibitor pembrolizumab in patients with advanced pancreatic adenocarcinoma was evaluated (NCT02620423). Of the 10 patients that could be evaluated for therapeutic effects, three achieved long-term benefits from the triple combination therapy; in detail, one patient achieved a partial response that lasted up to 17.4 months after treatment, and two patients achieved stable disease for 9.1 and 4.1 months (median OS of all patients enrolled in this study was 3.1 months) [134]. Post-treatment tumor biopsies of two patients showed increased CD8+ T cell infiltration in comparison to pretreatment biopsies, suggesting that the TME of desmoplastic tumors and the well-characterized immunosuppressive TMEs of pancreatic tumors could be overcome by triple combination therapy. Patients that derived clinical benefit in this study showed elevated expression of LILRA4 and ICOS, which are key factors in the IFN-γ response to reovirus infection, after pembrolizumab administration. This result shows that a virus-induced immune response in tumor tissues may be a critical factor in the synergy of OV with ICI therapy. Additionally, the trial compared peripheral blood at the time of pelareorep priming (cycle 1, day 8) versus that after administration of pembrolizumab (cycle 2, day 1). Greater clonal expansion of T cells during the pelareorep priming stage was associated with improved long-term patient outcomes compared to those achieved after pembrolizumab combination therapy, showing that the robust immune priming of TME by Reolysin was a prerequisite for patient response. Lastly, there was no immune-related toxicity following triple combination therapy, demonstrating that the addition of Reolysin to combined chemo- and immunotherapies is well tolerated [135].

### 3.6. Vesicular Stomatitis Virus

Vesicular stomatitis virus (VSV) belongs to the *Rhabdoviridae* family and is an enveloped, 11-kb, single-stranded RNA virus with a size of 70 nm [136]. Recombinant strains of VSV, such as VSV(Delta51)-NIS and VSV-IFNβ, have shown oncolytic properties in preclinical myeloma models [137,138] and in other cancer models [139,140,141,142]. VSV exhibits wide species tropism, allowing preclinical validation in immunocompetent rodents [143]. Furthermore, Stojdl et al. have demonstrated attenuated versions of VSV, such as AV1 or AV2, to have oncolytic properties, as these viruses are capable of selective replication in IFN-deficient cancer cells, while normal cells have an intact IFN response to resist viral infection [144].

#### VSV-hIFNβ-NIS + Avelumab and VSV-hIFNβ-NIS + Pembrolizumab

VSV-hIFNβ-NIS is an oncolytic VSV expressing human IFN-β and sodium iodide symporter (NIS) reported to induce rapid and potent tumor remission following systemic therapy [145,146]. Several preclinical investigations of VSV-hIFNβ-NIS have been performed in acute myeloid leukemia (AML) models, as recent preclinical and clinical studies have demonstrated the significant therapeutic value of OV against hematologic malignancies [145,147,148]. In particular, the virus has demonstrated the potential to induce an antitumoral immune response in AML as a monotherapy or in combination with the PD-L1 inhibitor Ab, made evident by an increase in tumoral CD4^+^ and CD8^+^ T cell infiltration. Furthermore, Shen et al. demonstrated the depletion of NK or CD8^+^ immune cells to result in ineffective combination therapy, suggesting the need for induction of cellular antitumor immune responses to improve survival [146].

These preclinical findings led to the inclusion of VSV-hIFNβ-NIS in two combination phase I trials: one trial combined it with an anti-PD-L1 Ab, avelumab, in patients with refractory metastatic solid tumors (NCT02923466), while the other combined it with an anti-PD1 Ab, pembrolizumab, in patients with refractory non-small-cell lung carcinoma (NSCLC) or head and neck squamous cell carcinoma (NCT03647163). As both trials are in the recruitment phase, no data have been published, and further investigation is needed on the safety profiles and antitumor effects of the combination trials [135].

### 3.7. Maraba Virus

Maraba virus (MV) is a member of the Rhabdovirus family with an RNA genome [149]. MVs are relatively simple viruses but possess a number of properties that suggest their use as OV agents. For example, MVs are rarely associated with human disease, and pre-existing immunity against MV is rare in most populations [148]. In addition, the entire life cycle of MV occurs in the cytoplasm, preventing the opportunity for insertional mutagenicity [150]. Furthermore, despite the small genome size of the virus at 11 kb, MVs can be genetically manipulated for transgene insertion [150].

#### MG1-MAGEA3 + Pembrolizumab and MG1-E6E7 + Atezolizumab

Preclinical development of MV as an OV agent began with a genetically modified version of the wild type, MG1. Bourgeois-Daigneault et al. have shown that neoadjuvant treatment with MG1, prior to surgical tumor resection, improved survival and decreased both the size and number of subsequent lung metastases in triple negative breast cancer (TNBC) models [151]. Further, Zhang et al. demonstrated the intrinsic ability of attenuated MG1 to induce an NK cell-dependent antitumor response in a lung metastases model of melanoma [152]. Bourgeois-Daigneault et al. demonstrated that the antitumor immune response-inducing properties of MG1 can be further augmented by combination with anti-PD-1 or -CTLA-4 therapy, as the combination therapies induced more robust tumor growth inhibition than did the monotherapy controls [151].

MG1 has been investigated in clinical trials as an oncolytic cancer vaccine expressing MAGE-A3, which is a known tumor antigen selectively expressed in several types of tumors, such as NSCLC, melanoma, and certain hematological malignancies [89]. Specifically, preclinical work has demonstrated that primates primed with non-replicating Ad expressing MAGE-A3 (AdMA3) and boosted by oncolytic MG1-MAGEA3-induced MAGE-A3-specific CD4+ and CD8+ T cell activation [150]. This dual viral approach was investigated further as the basis of the first MG1-MAGEA3 clinical trial (NCT02285816), where it was administered with and without an AdMA3 in incurable MAGE-A3-expressing solid tumors. Interim reports of this trial have demonstrated AdMA3 followed by MG1-MAGEA3 to induce a potent antitumor immune response, confirming preclinical evidence reported by Pol et al. [153]. One potential concern for this strategy is that the function of MAGE-A3, along with those of similar family members MAGE-A2 and -A9, is relatively unknown and has conflicting reports. Some groups have demonstrated pro-tumorigenic effects and association with poor overall survival rate of antigen overexpression, while others have focused on the strong antitumor immune response-inducing potential of the antigen in vaccination strategies [153,154,155]. Despite the potential oncogenic activity of MAGE-A3, it is worth noting that recombinant MAGE-A3 protein administration in a large-scale randomized phase III trial was shown to be safe in NSCLC patients [156]. Due to these reasons, more in-depth safety analysis upon completion of the two phase I trials examining the therapeutic effect of MG1-MAGEA3 is eagerly awaited. MG1-MAGEA3 is also being investigated in combination with a PD-1 inhibitor, pembrolizumab, in patients with first standard therapy-resistant NSCLC (NCT02879760) or metastatic melanoma and cutaneous squamous cell skin cancer (NCT03773744). The NCT02879760 trial is underway, but no data have been reported, while the NCT03773744 trial is not yet recruiting participants.

Another MG1 oncolytic vaccine expressing an attenuated version of human papillomavirus (HPV) E6 and E7 proteins (MG1-E6E7) as therapeutic transgenes is under clinical investigation [157]. In a similar strategy to the MG1-MAGEA3 + AdMA3 tumor vaccination approach, tumors are first primed with replication-incompetent Ad expressing HPV E6 and E7 (AdE6E7), followed by the administration of oncolytic MG1-E6E7 and the anti-PD-L1 inhibitor atezolizumab in patients with advanced or recurrent HPV-associated tumors (NCT03618953). No data have been published, and further investigation is needed on the safety profiles and antitumor effects of the combination trial [158].

### 3.8. Poliovirus

Poliovirus belongs to the *Picornaviridae* family and has a 7.5-kb genome [159]. While the poliovirus is naturally oncolytic [160], it has been shown to be potently tropic toward CD155, an immune checkpoint molecule expressed in a wide range of malignant cells of solid tumors [161]. The pathogenicity, such as neurovirulence, of wild-type poliovirus is well known and must be adequately addressed before use as a therapeutic agent [161]. An example of an attenuated poliovirus is the Sabin vaccine, which was genetically modified to reduce its poliomyelitis effects while retaining its oncolytic activity [161].

#### PVSRIPO + Nivolumab and PVSRIPO + Atezolizumab

PVSRIPO is an oncolytic Sabin type 1 poliovirus that had an endogenous internal ribosome entry site (IRES) genetically replaced with a foreign IRES of human rhinovirus type 2 (HRV2) to attenuate the neurovirulence [161,162]. Preclinical studies of PVSRIPO demonstrated two notable findings: first, a single injection of the virus was equally effective in decreasing tumor burden compared to anti-PD-1 or anti-PD-L1 treatments in TNBC models [163]. Second, PVSRIPO in combination with anti-PD-1 or anti-PD-L1 therapy improved tumor growth inhibition compared to checkpoint blockade monotherapies [163].

Based on these results, PVSRIPO was evaluated for its safety as a monotherapy in a phase I trial in patients with RGBM (NCT01491893). A total of 61 patients received a dose of PVSRIPO without evidence of viral neuropathogenicity or virus shedding. However, 19% of the patients in the dose-expansion phase experienced grade 3 or higher PVSRIPO-related adverse events. The survival rate of patients treated with PVSRIPO was higher at 36 months after treatment compared to historical controls [164]. These results were the basis for two combination trials with PVSRIPO and ICIs. One of these trials investigated the combination of the virus with an anti-PD-1 inhibitor, nivolumab, in patients with anti-PD-1 resistant melanoma (NCT04125719), while the other investigated the combination of PVSRIPO with a PD-L1 inhibitor, atezolizumab, in patients with RGBM (NCT03973879). However, both trials were withdrawn, although they are expected to be resubmitted, and no data have been published.

### 3.9. Newcastle Disease Virus

Newcastle disease virus (NDV) is a neurotropic virus with a 15-kb, single-stranded RNA genome and belongs to the *Paramyxoviridae* family [165]. NDV has been shown to be an effective oncolytic agent, reported to replicate up to 10,000 times faster in human cancer cells than in most normal human cells [166]. Further, Schirrmacher et al. have shown NDV-treated tumors to be infiltrated with T cells secreting IFN-γ [167]. In addition, Koks et al. reported NDV infection to induce long-term survival and tumor-specific immunity in an orthotopic glioblastoma multiforme model in immunocompetent mice, while no therapeutic effects were seen in immunodeficient Rag2 (−/−) mice or mice depleted of CD8^+^ T cells [168].

A preclinical study revealed that intratumoral administration of NDV and systemic CTLA-4 blockade induced inflammatory responses and antitumor effects in both local and distant tumors via the induction of systemic and tumor-specific immune responses. Zamarin et al. also reported that localized administration of NDV induced a systemic tumor inflammatory response, as evidenced by the increased infiltration of activated effector T cells in non-injected distant tumors [165]. In addition, the authors found combination therapy of localized NDV and systemic CTLA-4 or PD-1 blockade to produce antigen-dependent tumor rejection in a tumor rechallenge model [166]. Zamarin et al. developed an NDV expressing an ICI molecule, ICOSL (NDV-ICOSL), which in combination with anti-CTLA-4 therapy induced a more effective rejection of distant tumors than NDV monotherapy, leading to the long-term survival of the majority of mice and providing protection against tumor relapse [169].

#### MEDI5395 + Durvalumab

MEDI5395 is a recombinant NDV encoding a GM-CSF transgene. MEDI5395 in a preclinical study was shown to preferentially replicate in tumor tissues, elevate PD-L1 expression, and induce an antitumor immune response [170]. Further, MEDI5395 treatment increased T cell recruitment and expression of IFNγ-inducible genes in vitro, suggesting immune activation of the TME. For these reasons, MEDI5395 has been recently entered in a phase I trial in combination with the PD-L1 inhibitor durvalumab (NCT03889275). However, as the trial is still in the recruitment phase, no data have been published, and further investigation is needed on the safety profiles and antitumor effects of the combination therapies.

## 4. Conclusions

The use of IO has changed cancer treatment in recent years. Despite ICI revolutionizing the cancer therapy field, it is largely ineffective in patients with cold tumors. To address this limitation, OVs in combination with ICIs can be a strategy to address the inefficacy of ICI against cold tumors, as these viruses can inflame the TME into a more immunologically favorable environment for cancer immunotherapeutics. The number of clinical trials investigating combinations of OVs and ICIs continues to rise, and most of the available results from these trials have demonstrated promising therapeutic potentials with good safety profiles. Other immunotherapeutics (like CAR-T and ICI) in combination with ICIs are well known to compound adverse events, whereas OVs do not, and this superior safety profile is particularly promising in IO. Due to the recency of many of these clinical trials, in-depth results are not available to the public, but many of the interim and preclinical results indicate strong synergism of the combination therapy approaches, as evidenced by increased migration of antitumor immune effector cells to tumor tissues and elevated expression of antitumor cytokines. The detailed results of these studies are highly anticipated, as they will provide much needed insights that will be critical to maximizing the therapeutic potential of OV and ICI combination therapies for treatment of intractable cancers. With increasing numbers of OV pipelines and ICIs entering clinical development, there is a strong potential for this strategy to revolutionize cancer treatment in the near future.

## Figures and Tables

**Table 1:** List of ongoing combination therapy clinical trials with oncolytic viruses and immune checkpoint inhibitors.

Oncolytic Virus	Variant	Modification	Combination Agent	Tumor Type	Identifier
Adenovirus	CG007	GM-CSF	Pembrolizumab	Bladder cancer	NCT04387461 Phase II
	Ads/HSV-tk	HSV-tk	Pembrolizumab	TNBC, NCLC	NCT03004183 Phase II
	Enadenotucirev	A11/Ad3 Chimeric backbone	Nivolumab	Advanced epithelial tumors	NCT02636036 Phase I
	Tasadenoturev	Delta-24-RGD	Pembrolizumab	Recurrent glioma	NCT02798406 Phase II
	ONCOS-102	GM-CSF	Pembrolizumab	Unresectable melanoma	NCT03003676 Phase I
	LOAd703	4-1BBL+ CD40L	Atezolizumab	Malignant melanoma	NCT04123470 Phase I/II
	Telomelysin	hTERT	Pembrolizumab	Advanced solid tumor	NCT03172819 Phase I
	Telomelysin	hTERT	Pembrolizumab	Esophagogastric adenocarcinoma	NCT03921021 Phase II
Herpes Simplex Virus-1	Imlygic	GM-CSF	Ipilimumab	Melanoma	NCT01740297 Phase I/II
	Imlygic	GM-CSF	Pembrolizumab	Stage IIIB–IV melanoma	NCT02263508 Phase III
	Imlygic	GM-CSF	Pembrolizumab	Stage III–IV melanoma	NCT02965716 Phase II
	Imlygic	GM-CSF	Pembrolizumab	HNSCC	NCT02626000 Phase I
	Imlygic	GM-CSF	Pembrolizumab	Sarcoma	NCT03069378 Phase II
	Imlygic	GM-CSF	Pembrolizumab	HCC, liver metastases	NCT02509507 Phase I
	Imlygic	GM-CSF	Nivolumab + Trabectedin	Sarcoma	NCT03886311 Phase II
	Imlygic	GM-CSF	Atezolizumab	TNBC, CRC	NCT03256344 Phase I
	HF10	Spontaneously mutated	Ipilimumab	Melanoma	NCT03153085 Phase I
	HF10	Spontaneously mutated	Ipilimumab	Melanoma	NCT02272855 Phase II
	HF10	Spontaneously mutated	Nivolumab	Stage IIIB, IIIC and IVM1a melanoma	NCT03259425 Phase II
Vaccinia Virus	Pexa-Vec	Human GM-CSF + β-galactosidase	Ipilimumab	Advanced solid tumors	NCT02977156 Phase I
	Pexa-Vec	Human GM-CSF + β-galactosidase	Durvalumab	CRC	NCT03206073 Phase I/II
	Pexa-Vec	Human GM-CSF + β-galactosidase	Nivolumab	HCC	NCT03071094 Phase I/II
Coxsackievirus	CAVATEK	Unmodified	Ipilimumab	Uveal melanoma	NCT03408587 Phase I
	CAVATEK	Unmodified	Ipilimumab	Melanoma	NCT02307149 Phase I
	CAVATEK	Unmodified	Pembrolizumab	Melanoma	NCT02565992 Phase I
	CAVATEK	Unmodified	Pembrolizumab	NSCLC and bladder cancer	NCT02043665 Phase I
Reovirus	Reolysin	Unmodified	Pembrolizumab	Pancreatic cancer	NCT02620423 Phase I
Vesicular Stomatitis Virus	VSV- IFNβ-NIS	IFNβ and sodium importer gene	Pembrolizumab	NSCLC and HCC	NCT03647163 Phase I
	VSV- IFNβ-NIS	IFNβ and sodium importer gene	Avelumab	Refractory solid tumor	NCT02923466 Phase I
Maraba Virus	MG1-MAGEA3 + Ad MAGEA3	MAGE	Pembrolizumab	NSCLC	NCT02879760 Phase I/II
	MG1-E6E7	HPV E6 and E7	Atezolizumab	HPV-associated cancers	NCT03618953 Phase I
	MG1-MAGEA3 + Ad MAGEA3	MAGE	Pembrolizumab	Metastatic melanoma	NCT03773744 Phase I
	PVSRIPO	Human rhinovirus type 2 (HRV2) IRES	Nivolumab	Melanoma	NCT04125719 Phase I
	PVSRIPO	Human rhinovirus type 2 (HRV2) IRES	Atezolizumab	RGBM	NCT03973879 Phase I/II
Newcastle Disease Virus	MEDI5395	GM-CSF	Durvalumab	Advanced solid tumors	NCT03889275 Phase I

## Abbreviations

IO	Immuno-oncology
ICI	Immune checkpoint inhibitor
IgG1	Immunoglobulin G
CTLA-4	Cytotoxic T-lymphocyte antigen-4
TME	Tumor microenvironment
OV	Oncolytic virus
Ab	Antibody
PD-1	Programmed death 1
PD-L1	Programmed death-ligand 1
irAE	Immune-related adverse events
TIL	Tumor infiltrating lymphocyte
HMGB-1	High-mobility group protein box 1
TIM-3	T cell immunoglobulin and mucin domain 3
APC	Antigen-presenting cells
IFN- γ	Interferon gamma
IL-4	Interleukin
DC	Dendritic cells
LAG-3	Lymphocyte-activation gene 3
MHC	Major histocompatibility complex
Treg	Regulatory T cells
RGBM	Recurrent glioblastoma
Gal-9	Galectin-9
CEACAM1	Carcinoembryonic antigen-related cell adhesion molecule 1
PS	Phosphatidylserine
TIGIT	Tyrosine-based inhibition motif domain
VISTA	V-domain Ig suppressor of T cell activation
PAMPS	Pathogen-associated molecular patterns
TAA	Tumor-associated antigens
DAMPS	Danger-associated molecular patterns
IL-12	Interleukin 12
GM-CSF	Granulocyte-macrophage colony-stimulating factor
HSV	Herpes simplex virus
MAGE-A3	Melanoma-associated antigen 3
NY-ESO-1	New York esophageal squamous cell carcinoma 1
TNF-α	Tumor necrosis factor-alpha
IL-2	Interleukin 2
hTERT	Human telomerase reverse transcriptase
MART	T cell-recognized melanoma antigen
DLT	Dose-limiting toxicity
ICAM	Intercellular adhesion molecule 1
DAF	Decay-accelerating factor
NIS	Sodium iodide symporter
NSCLS	Non-small-cell lung carcinoma
AML	Acute myeloid leukemia
TNBC	Triple negative breast cancer
IRES	Internal ribosome entry site
NDV	Newcastle disease virus
ICOS	Inducible co-stimulator
